# Using a quality improvement framework to evaluate the feasibility of implementing a patient-reported outcome measure for recovery in an office-based treatment setting for opioid use disorder

**DOI:** 10.1186/s13722-025-00632-4

**Published:** 2025-12-22

**Authors:** Elisabeth Okrant, Sharon Reif, Genie L. Bailey, Constance M. Horgan, Margaret T. Lee, Madeline A. Brown, Blaire L. Simas, Karen A. Alfaro, Grant A. Ritter

**Affiliations:** 1 Institute for Behavioral Health, Heller School for Social Policy & Management, Brandeis University, 415 South Street, Waltham, MA 02453 USA; 2 SSTAR, 386 Stanley Street, Fall River, MA 02720 USA; 3 Department of Psychiatry and Human Behavior, Warren Alpert Medical School, Brown University, 22 Richmond Street, Providence, RI 02903 USA

**Keywords:** Feasibility, Implementation, Patient-reported outcome measures, PROM, Recovery, Opioid use disorder, Substance use disorder, Change management, Quality framework, Culture

## Abstract

**Background:**

Patient-reported outcome measures (PROMs) offer a way to track patient recovery from substance use disorder (SUD) and make clinical decisions more effective and efficient. PROM implementation in the context of the SUD population is not yet well understood, but recovery is the type of outcome that requires self-report. While studies have found PROM implementation feasible, an abundance of literature conveys the complexities of implementing PROMs in routine clinical care. We report here on the feasibility of incorporating a PROM for SUD recovery within a buprenorphine program for people with opioid use disorders at a federally qualified health center.

**Methods:**

We describe the challenges and distill learnings gained from the feasibility study of the Response to Addiction Recovery (R2AR) PROM instrument that was tested for validity and feasibility with staff (nurse care managers; NCMs) and clients of an office-based addiction treatment program. We assessed the R2AR in terms of clinical workflow and value to clients and clinicians within a fully remote research study which influenced the findings regarding workflow and value. We used the IHI root cause analysis framework to organize results, capturing learning relating to culture, systems, process, people, and environment.

**Results:**

The three most significant barriers to implementing R2AR included the need to identify a champion and change agent, educate clients and providers to disrupt cultural norms, and use the right care team member to implement the intervention. These barriers were amplified by the limited time that NCMs had available to engage with clients and the fact that R2AR’s psychosocial perspective was outside of their medication management emphasis.

**Conclusions:**

Implementing a clinically-based PROM requires clients and providers to alter the way in which they currently behave. A new tool alone cannot change the culture that ascribes provider and client roles within healthcare. The culture of patient-centered care must precede PROM adoption for effective changes to occur. Systems, such as the EHR, must mandate PROM administration to allow workflows to adjust to new processes, and to convey its import as part of clinical care. Until systems are updated, and large-scale PROM adoption becomes ubiquitous, PROMs can also play an important role in supporting self-management and shared decision making.

**Trial registration:**

This study is registered at ClinicalTrials.gov (https://clinicaltrials.gov/, NCT05388045, registered April 14, 2022).

## Key messages regarding feasibility

1. Successful adoption and use of patient-reported outcome measures (PROMs) in clinical workflows (i.e., cancer treatment, knee replacement) requires a culture of shared decision making and mutual respect, whereas people with substance use disorders are traditionally disempowered as providers drive what happens in formal addiction treatment.

2. While patients and providers conceptually agreed that the addiction recovery PROM, Response to Addiction Recovery (R2AR), was a benefit for recovery planning and patient centeredness, actual adoption was thwarted by implementation barriers that are common to other change management efforts in quality improvement.

3. The findings suggest that more time spent during the study design phase focused upon culture, including roles and responsibilities of patients and providers, is necessary for PROM adoption, as are the systems and processes that facilitate adoption as part of best practices.

## Background

Measuring outcomes is critical to evaluating quality of care. Highlighting recovery as a formal outcome of substance use disorder (SUD) treatment has been elusive, in part because recovery remains a complex and debated construct without a single consensus definition [1–5]. Healthcare fields [6, 7], including SUD [8, 9], have turned to patient-reported outcomes (PROs) as a means for measuring subjective outcomes that heretofore have been unmeasurable because they use data collected directly from clients about factors that impact health and quality of life [10]. Furthermore, PROs reflect a growing movement toward patient-centered care, whereby clients provide feedback on their health state and treatment goals (e.g., experiences and outcomes) without interpretation from providers, which occurs with clinician-reported outcomes [11].

PROs can therefore support active patient-centered participation in care, such as enabling patient directed goals, planning, and care decisions [9]. Moreover, self-report via patient-reported outcome measures (PROMs) supports the clinical monitoring and self-management of incremental recovery progress and the direct measurement of intervention on health improvement [12]. PROMs offer a way to track patient recovery journeys and make clinical decisions more effective and efficient. While PROM implementation in the context of the SUD population is not yet well understood [13], recovery is the type of outcome that must rely on self-report. Measuring recovery through a PROM could facilitate the way that patients and providers assess the quality of care.

### Barriers to PROM implementation

PROMs should be precise and reliable for patients and providers to recognize their value [14]. Moreover, if they are to be adopted successfully as part of clinical practice, they cannot create undue burden for either patients or providers because the pain points of implementation will outweigh the perceived benefits [15]. Only when adopted uniformly can an instrument be successfully used as standard care to measure clinical quality and guide recovery efforts. While feasible [16], an abundance of literature conveys the complexities of implementing PROMs as part of routine clinical care. Barriers can lead to suboptimal data collection and impede clinical use [8]. Decreased psychometric accuracy also occurs with repeat administrations and survey fatigue for both patients and providers who value the results less over time [7]. Common barriers include the inability to embed PROMs into clinical workflows due to time constraints [17, 18]; the lack of appropriate staff and patient training [19, 20]; inadequate information technology infrastructure to support data collection [21]; and the inability to incorporate results as part of clinical encounters [20, 21].

We report here on the feasibility of incorporating a PROM for SUD recovery, the Response to Addiction Recovery (R2AR) [3] within a buprenorphine program for people with opioid use disorders at a federally qualified health center. Because feasibility studies have fundamental crossover with change management and quality improvement (QI) frameworks – in their ability to reveal barriers to adoption of best practices [22] – we employed these concepts and categorizing principles to interpret the results of our feasibility study.

## Methods

We provide here information on the root cause analysis framework we used, the goals of the feasibility study, and the clinical setting, including staff and clients, where the feasibility study of a recovery PROM was conducted. The development of this PROM is described elsewhere [3], and the aim to assess the validity of this PROM as a recovery measure is underway and will be reported elsewhere. We used the Standards for Quality Improvement Reporting Excellence (SQUIRE) Guidelines, V.2.0 [23] as a reporting standard which most closely reflected this design and analysis of this feasibility study. The study was approved by the Brandeis University Institutional Review Board.

### Quality improvement root cause analysis framework

Implementation feasibility studies aim to understand strategies to improve adoption because change (i.e., process, clinical guidelines, etc.) has downstream effects across the healthcare ecosystem. We chose the IHI quality improvement root cause analysis framework employed in healthcare which provides a systematic approach to understanding the reason an issue or error occurs [24, 25]. Using this approach, we categorize barriers to feasible implementation into themes: culture, systems, process, people, and environment. We provide here an overview of the types of barriers to feasibility of PROM implementation within each category.

#### Culture

Culture is the shared understandings and beliefs that shape how work is performed and replicated [26]. Importantly, culture can be a barrier if the change itself is not compatible with prior concepts that governed how work was performed [27]. Therefore, PROMs that encourage patient-directed goal setting, and measure recovery based upon an individual’s notions, challenge cultural norms within the practice of medicine, where providers typically guide treatment decisions based upon their expertise [28]. This provider-centric approach is emphasized with conditions such as SUD, when clients are particularly vulnerable because of self- and external stigmas, as well as the real or perceived challenges related to decision making because of lasting influences that substance use has on cognitive abilities [29, 30]. Shared decision-making models in SUD facilitate building autonomous, personalized care plans and improve adherence to treatment [31–33]. Yet, tools to standardly collect data on recovery pathway preferences and monitor changes, as a provider-patient team, are not often employed, despite shared decision-making leading to greater trust in providers and longer-term recovery outcomes. We conducted focus groups and interviews with both providers and patients as part of our study to develop learnings about the degree to which R2AR supports shared decision making.

#### System

A change to staff processes requires systemic support via technology to ensure that new operations and automatic fail-safes, such as alerts and reminders, are embedded in workflows [34]. The electronic health record (EHR) is the best mechanism to ensure that patient safety and quality protocols are followed because, through automation and auditable events, it codifies evidence-based practices and ensures compliance via standardized clinical workflows [35]. Activities outside of the EHR are not transparent or standardized, therefore result in uneven and inconsistent practices and reporting because their omission from the EHR signals lower priority [36]. Moreover, EHRs were built to align with “objective” healthcare data, such as lab tests. PROM adoption would require system changes to allow input of more fluid, less objective data in a standardized manner.

#### Process

Longitudinal data collection for tracking trends requires processes that support ongoing, repeated, and standardized administration of outcome instruments [37]. However, healthcare delivery is often fragmented, leading to a lack of care continuity. Therefore, repeated administration of a PROM, intended to develop a trajectory of progress, is incongruous with current service delivery models that emphasize acute care delivery and do not facilitate chronic disease management and prevention.

#### People

Roles, responsibilities, staffing levels, and agency impact adoption of new interventions which therefore benefit from the advocacy of both change agents (front-line influencers who encourage adoption because the change influences how their work is performed, too) and champions (leaders who sponsor the adoption by leading through change, deploying resources, and highlighting wins). Change agents are necessary because the known way of doing work is difficult to disrupt without justification that it is worthwhile. They can help reduce emotional unease among a group adopting unfamiliar processes by offering perspectives to influence the belief that benefits outweigh costs [38]. Since change agents often experience the same challenges as their colleagues, they are in a better position relative to leadership to help staff understand the purpose of the change and overcome negative attitudes or resistance. A champion is also influential in the change process by addressing barriers and promoting alignment across the organization. For example, champions might work cross-functionally to address downstream or collateral effects from the change on other aspects of the organization. While these roles are often fulfilled by different people, they could be the same individual, especially in smaller organizations.

#### Environment

Successful intervention planning accounts for the context in which it is deployed Real-world implementation should include scenario planning and consider possible future outcomes, including risk mitigation strategies. For example, COVID-19 changed the healthcare landscape in ways that had not been anticipated. Its impact changed many of the taken-for-granted aspects of the environment in which interventions were deployed, such as workflows supported by brick-and-mortar facilities and in-person care.

### Study objective

Given the challenges and barriers of using a PROM, we developed and tested the feasibility of implementing a PROM to measure recovery in a clinical setting. Our research team developed the Response to Addiction Recovery (R2AR), with input from clients and providers [3]. It uses two prompt questions (“how much do you agree with ____?” and “how important is ___ for you to work on now?”) with 19 multidimensional items that represent elements of the recovery construct [3]. As a team of clinicians, researchers, and quality experts, we are acutely aware of the challenges that workflow poses to implementing new treatment approaches or interventions. We thus embarked on a feasibility study to examine the use of the R2AR for data collection and quality measurement. We posited that data from the R2AR would be clinically significant enough to be perceived as a benefit to both the clients and providers, thereby mitigating many potential barriers to acceptance.

With the potential barriers in mind, we developed a pilot clinical trial to validate and assess the feasibility of implementing our R2AR recovery PROM at a federally qualified health center among clients receiving buprenorphine to treat opioid use disorder (OUD). We followed the National Quality Forum guidance on PROM development [39] to test the feasibility of implementing into clinical practice early in its development.

### Setting

Our feasibility study was conducted with staff and clients of a community treatment center in Fall River, Massachusetts, which has an office-based addiction treatment (OBAT) program embedded in its federally qualified health center. The participating clients were enrolled into the OBAT program where buprenorphine is prescribed. Their primary clinical contact during their maintenance phase was with the nurse care managers who provided medication management visits; client contact with the buprenorphine prescriber usually occurred every 4–8 weeks. The medical director of this program collaborated throughout the study design, implementation, and analysis of results. We anticipated that active participation by program leadership who served as a champion would mitigate some implementation barriers. An on-site research assistant (RA) was hired to assist with recruitment, screening, tracking, and scheduling of clients and communication with nurse care managers.

### Clients

Participants were clients receiving buprenorphine through the OBAT program for at least 30 days and not longer than 3 years. The length of time on buprenorphine was selected to enlist a study population that we hypothesized would be a representative sample of early and active recovery, resulting in variation in the PROM scores, along with the ability to track our hypothesis for the validity study that people in early recovery would have greater changes in PROM scores than people in longer term recovery. We collected informed consent and provided clients with incentives for completing the R2AR and the associated research assessments. Clients were paid $20 for each time point they participated, for a maximum of $60. We consented *N* = 110 clients, of which *N* = 94 completed the first R2AR and *N* = 27 completed the second R2AR.

### Clinicians

Participating clinical staff were the nurse care managers (NCMs) who were registered nurses in the OBAT program. The number of participating NCMs varied over time due to staff turnover, which was exacerbated during the pandemic: 7 were initially engaged, 3 left and were replaced, and 5 were added, for a total of 15 NCMs involved over time. The implementation of the R2AR was considered a quality improvement activity. NCMs were consented but the OBAT leadership expected them to participate by engaging with the R2AR as part of their clinical practice. As an incentive, clinicians were offered $50 after discussing 5 R2AR instruments with their clients, with the expectation that it would encourage ongoing use of the R2AR once it was familiar.

### Feasibility study design

Prior to the start of the R2AR implementation, clients and NCMs were actively involved in refinement of its questions [3]. We anticipated that this input would increase the value of the instrument for the NCMs to use in their clinical work. The RA supported all research-related contact with the clients, collection of research assessments, and administrative support for NCMs whose clients who were in the study. The original study design called for clients to complete the R2AR and research assessments at baseline and at 3- and 6-months, timed to match an in-person OBAT visit by the client. Study recruitment was scheduled to begin in early 2020, with all activities intended to take place in person. With the onset of the COVID-19 pandemic, the treatment center pivoted to the new environment dominated by remote online communications. Treatment moved to a telehealth model for OBAT clients, and this model was largely maintained throughout the study period.

Client recruitment was thus done remotely (e.g., with letters, texts, and phone calls). The R2AR and research assessments were collected by computer or smartphone via an online survey tool (Qualtrics) and in one combined assessment. Interaction between the RA and the clinicians was also largely by email, text and phone. We offered phones for use during the study period for clients who did not have them, or whose data plans were limited, to reduce barriers due to technology-enabled communication; phones were accepted by 12 clients. To facilitate study adherence and promote completion of the follow-up R2AR, the study design called for the RA to securely email NCMs their clients’ R2AR responses the morning of their appointments for use during their visit. In addition, we asked NCMs, by a 2-question emailed survey link after the visit, if they talked about the R2AR with their clients and if they considered it helpful in the clinical interaction. We decided early in the study to drop the third data collection point. Clients were recruited from April 2022 through August 2023.

A mid-study focus group was conducted on-site to solicit feedback from the NCMs on the survey format and study procedures. Following this focus group, the order of items on the R2AR was reworked to improve efficiency, flow, and clarify the difference between the two question prompts. We also conducted three interviews with clients at the midway point to get feedback on the use of R2AR as part of their treatment process.

The feasibility study assessed the R2AR in terms of clinical workflow and value to clients and clinicians. We also considered the delivery of care and research methods using a decentralized approach, particularly because the definition of care and services are no longer tethered to brick and mortar. We organize our results by the root cause analysis QI framework [25], capturing learning relating to culture, systems, process, people, and environment.

## Results

### Culture

Our request to use the R2AR to measure recovery progress and guide clinical decisions was perceived as inconsistent with the culture of care at the OBAT in which we conducted the study. Following the change management literature, the key question we wanted to address was whether a PROM instrument that enabled providers and clients to celebrate wins together and review recovery through the prism of progress and change could fit in or alter the recovery treatment culture in such a way that it would encourage clients to complete the survey and lead providers to use it during the course of treatment The answer we found was that developing protocols for process adoption was not adequate for change management, but results also suggested that a champion/change agent could help and lead to at least some success in implementation.

### Systems

Because we were conducting a feasibility study, we could not embed R2AR in the EHR. Therefore, both providers and clients were asked to complete tasks outside of the standard platforms, which resulted in greater burden for both groups as reported in both provider and client focus groups. Furthermore, the insights from the R2AR had to be independently determined by the NCMs and were not automatically generated or returned to clients and providers through the usual technological interface used for healthcare.

Like providers, clients reported that they did not experience the PROM as integrated within clinical practice because they completed it through an online survey tool prior to their appointment. The midpoint interviews with several clients revealed that R2AR was not discussed during their visits with NCM, nor was the intent of the tool discussed – to guide client directed recovery. Nevertheless, we received 56 NCM responses to the two questions asked immediately following the OBAT visit where the R2AR was used: 98% reported that they talked with their clients about the R2AR, and 71% found it useful, with another 20% who found it somewhat useful.

While nearly all responses from NCMs suggested that they did use the R2AR, we received provider input on only 56 of 127 total visits for which an R2AR was available. We do not know if the lack of response by NCMs was because they did not open the email or did not engage with the R2AR at all. Systems, on the other hand, would have time stamped and automated processes to ensure workflows are completed according to best practices.

### Process

Only one-third of clients completed a second R2AR. The plan to administer the PROM in three-month increments was determined during the design phase, in conjunction with the NCMs, as the amount of time that we would expect to observe changes in scores on the recovery items given typical appointment cadences. However, the feedback we received from NCMs during the study suggested that repeat administration was hindered by asynchronous data collection issues and other feasibility factors.

### People

NCMs have limited time with clients and have honed and optimized their usual appointment times to address clinical issues. Therefore, they regarded the output of the R2AR, which is primarily psychosocial, as less important than the medication-related clinical issues that require their specialized attention during visits. As they explained in the midpoint focus group, they were unable to prioritize the R2AR despite recognizing that it offered recovery insights because the issues it addressed were outside their primary roles as clinicians who oversee and optimize medication management. NCMs have clinical requirements that are difficult to accomplish within 15–20 min appointments, leaving little time for additional activities. During the focus groups, NCMs reported that time was the primary barrier in their using the R2AR with the client. NCMs were not required, but rather incentivized and encouraged, to use the R2AR during patient visits. And, while most reported during the focus groups that the instrument was helpful in discussing recovery barriers with clients, NCMs did not implement the R2AR across all clients at all requested times. While they agreed the R2AR had value, time pressures prioritized completing other tasks.

We also found variation in the engagement with R2AR across NCMs, based on the 2-question email survey: two NCMs used the R2AR 15 or more times; two used it 6 or 8 times; five used it 2 or 3 times; and six did not report using it at all (some of whom may have been the most recently hired).

### Environment

The study design and workflow of our study had to be changed to adjust to the COVID-19 public health emergency, in particular given the pivot of both treatment and research to remote care delivery. At that point, we learned that some clients did not possess the technological dexterity [40] to use the online survey tool to complete the R2AR and research questionnaires and required that the RA guide them through survey completion.

We experienced a number of challenges due to our changed study design and remotely administering the R2AR. NCMs and clients were adjusting in real time to virtual treatment, such as logging into secure information technology and communications systems [41]. Many clients did not have video capabilities for telehealth, limiting clinician’s abilities to use physical cues about clients’ well-being [42]. According to the NCMs, these new barriers resulted in even less time than before to accommodate additional requests like R2AR.

## Discussion

Although recovery is a core concept in addiction treatment, measuring recovery (e.g., using a PROM) is still an innovative idea in addiction science and performance measurement [43, 44]. Recovery measurement has not yet been adopted regularly to guide clinical care or to evaluate quality of care [43]. Based on the results of this feasibility study, the three most significant barriers to implementing R2AR included the need to identify a champion and change agent on the ground, educate clients and providers to encourage the disruption of cultural norms, and use the right care team member to implement the pilot. These barriers were amplified by the limited time that NCMs had available to engage with the clients and the fact that the R2AR’s psychosocial perspective was outside of their medication management emphasis. The next phase of R2AR implementation must address these barriers given how critical they are in successful clinical adoption of a longitudinal PROM within SUD treatment.

Repeated time points for data collection are integral to the design of the R2AR as a clinical tool where, using the next R2AR at the point of care, clients and providers develop future-looking priorities and care plans. Ideally, the R2AR would link past, present, and future, whereby clients and providers discuss the previous period to review the clients’ priorities and results based on the plan developed collaboratively. Yet only one third of clients completed a second R2AR, thus we were unable to evaluate the impact of R2AR to achieve this goal. The linkage across time is fundamental to the use of the R2AR that seeks to embed lived experience into data collection. Future work should identify ways to improve repeat administration.

Our study found a clear benefit to having a champion (i.e., the OBAT medical director) to promote the benefits of using the R2AR as a tool for recovery and deflect the perception that R2AR use was outside of standard practice, but a change agent (i.e., a NCM) was needed to facilitate adoption and address barriers on the ground [45], especially with regard to education and training about how to use the PROM effectively and why “pains” of adopting the new process were worthwhile [46]. At this OBAT, NCMs are not independent decision makers and are not accustomed to acting autonomously; rather, they typically receive supervision or instructions from a prescriber concerning patient care delivery decisions. Yet, the role of change agent was difficult to encourage in the virtual space since NCMs were not sharing time and space that facilitate changing attitudes, such as staff room and in-person “hallway” discussions. Virtual weekly huddles with the NCMs and the OBAT medical director or the RA that focused on the R2AR and incorporating the psychosocial concepts of recovery likely could have kept the R2AR as a topic of ongoing conversation, inquiring what is and is not working. The change agent could have highlighted examples of recovery facilitated by the R2AR to persuade the NCMs of its value, making R2AR a regular topic at staff meetings.

We conducted the feasibility study to learn about the barriers of implementing recovery measures that allow us to measure clinical effectiveness and quality of care. We hypothesized that R2AR would facilitate leveling the hierarchical relationship between client and provider and support client-directed care but were unable to evaluate this assumption because NCMs did not implement it as intended. Instead, we learned that culture must support adoption and fidelity of instrument administration used for collecting data on recovery progress. While we were unable to assess whether the R2AR supported shared decision making, we did learn that a new tool alone cannot change the culture that constructs and reifies provider and client identities and ascribes scripts to how roles within healthcare are performed. The idea that the tool could improve visibility and transparency of quality of care by demonstrating how clients were improving in their recoveries may not have been valued significantly enough to increase R2AR adoption in the context of medication management visits. Further, the self-report aspect of a PROM has not yet been adopted by the field as a “true” assessment of SUD recovery [28], particularly due to the hierarchy between care providers and clients, which results in its perceived value to be discounted as compared to “objective” measures such as urine drug screens.

The culture of patient-centered care must precede PROM adoption for effective changes to occur [47, 48]. Moreover, once part of the standard of care, PROMs should be reported back to the patient to help them evaluate progress toward goals on specific items and guide care decisions. Simply put, clinical leadership are the drivers of cultural change that must mandate PROM administration and advocate for integrating these tools into EHRs to enable workflows that prioritize the administration and adoption of new processes as expected aspects of clinical care.

Just as providers required additional and ongoing education, clients also could benefit from education to better understand the value of the instrument [13]. Clients were introduced to the R2AR by an RA who was not expected to explain the virtue of the tool, but rather the role of study participation. Other educational pathways, such as explaining the value of R2R via group therapy, or watching a video filmed by the research team that role-played the benefits and explained how the tool was intended to be used, could have offered clients an understanding that promoted perceived value and use.

Neither clients nor NCMs assumed accountability for R2AR use in our study. Clients’ roles and expectations also had to shift for the provider-patient relationship to be rescripted as we thought might occur with the introduction of the R2AR [35, 49]. Not only did providers have to invite clients into the activity of assessing recovery status from the client’s point of view and participate in prioritizing those areas that are most important based on needs and preferences, but also clients also had to demonstrate agency [50]. While clients conveyed during the focus groups that they would like an instrument to share their feedback with providers, they were unlikely to insist on it during appointments according to NCMs.

As a result, with the choice of using R2AR information during the patient visit optional and with little understanding of its benefit, most NCMs determined that the additional burden could not be managed given time constraints. As an added complication, due to turnover in SUD treatment generally [51] and more so during the pandemic, many new staff were learning their roles and did not have the capacity to assume additional responsibilities. It is also likely that the pandemic affected NCMs, both personally and professionally [52], in ways that uniquely impacted each person’s ability to pivot within their jobs to incorporate new expectations that were considered to be peripheral. Work related to the feasibility study often had to be deprioritized in favor of completing requisite actions and workflows especially when the OBAT program was short-staffed. Some staff who had originally been trained on R2AR administration by the research team resigned during the study. Thus, trainings, huddles often used in clinical management, and ongoing oversight by a change agent at the clinical site might have improved compliance, but were less feasible in the context (i.e., the challenges imposed by pandemic-related changes).

Our study design was a strength that cultivated many take-aways about PROM feasibility. First, we used qualitative data from focus groups with NCMs and clients to update the items in the R2AR [3]. This allowed the research team to improve the precision of the instrument, and confirmed that clients and providers were interpreting the items similarly, thereby ensuring that patient-and-provider designed action plans were aligned to enable a shared understanding of the underlying areas for improvement. Secondly, we conducted a NCM focus group at the halfway point of the study to collect information about the feasibility and perceived value of including the R2AR as part of their practice. It led to a format change on the R2AR item prompts. The NCMs believed that this new cadence helped with the flow and allowed clients to think about each item and parse the questions more accurately, as well as lowered perceived burden. In our case, feasibility was improved by changing the PROM structure, which demonstrates the importance of field-testing instruments to mitigate barriers prior to implementation.

Previous literature suggests that resistance to change occurs when stakeholders do not perceive the value of change [53, 54]. While this is undoubtedly true in some circumstances, our study demonstrated that the value of change must be corroborated by the systems and processes designed to support healthcare operations [55]. Otherwise, any perceived value is trumped by the guardrails hardcoded into systems that govern how work is done, with the implied priorities and high value for those requirements. Certainly, this poses an existential problem to pilot feasibility studies that often lack resources or confirmatory analysis that support hardwiring the change prior to evidentiary support.

It is equally likely that the NCMs were misidentified in the study design as the care team members to be primarily responsible for incorporating R2AR information into patient care. We selected them given the OBAT structure that placed them in regular contact with clients. As previously mentioned, there was a lack of understanding among NCMs of the multiple purposes of the R2AR – to guide individualized care planning to improve outcomes as well as collect recovery data. Other members of the care team such as a recovery coach or care manager, who support the psychosocial aspects of recovery and are less constrained by clinical practice guidelines that govern treatment, might be more appropriate for a PROM intervention to generate shared decision making about recovery. Hence, the concordance between roles and activities are important to consider, especially when implementation in the future should better align with the systemic roles and responsibilities of providers.

Larger health systems with adequate resources to make systemic changes (in EHRs, etc.) might be better able to conduct pilot feasibility studies that reflect anticipated implementation in usual practice (i.e., by incorporating into the EHR) prior to advancing implementation to smaller, less resourced organizations once the evidence has been developed. Another possible solution is to pilot test PROMs as part of registries and clinical trials, and only later assess feasibility in real-world clinical practices. Also, to improve the generalizability of findings, we could use multi-site designs including large and small providers. Last, as a real-world implementation might, we could provide more training and education prior to launching the study and thus mitigate other root causes of adoption or failure to adopt.

### Limitations

We amassed learning from implementation of the R2AR in clinical practice that will impact subsequent PROM development, feasibility, and validation studies. Our learnings, we believe, are endemic to behavioral health and, as such, can be applied to future research and clinical studies. While our study design was strong, there were also several limitations.

While time was the greatest barrier for NCMs, the research team was unable to assess time burden on clients because they completed the R2AR online, and time calculations were only accurate it if was completed and submitted immediately; many participants did not do that. We conducted interviews to assess burden and satisfaction, but because of the online data collection approach clients were unable to distinguish the R2AR from the other research questionnaires they completed for the validation study. Therefore, it is difficult to isolate patient-interpreted burden for the R2AR itself.

A fully remote patient population required modalities that would enable outreach with clients off-site which was a greater challenge and required multiple approaches. One method that we believe yielded more participants was use of physical recruitment letters to reduce cold calls, which increased the likelihood of clients responding to phone calls or texts from the RA, and thus being consented into the study. While communication strategies today often prioritize digital methods, the patient population and their preferences are important to determine when designing recruitment methods. For this population, physical letters proved effective given the context and population of interest. Remote treatment, however, created barriers to retention (i.e., completing a second R2AR). Importantly, there can be unintended consequence of administering a tool digitally if clients do not possess the adequate reading skills or digital literacy to complete it [56].

We consider the feasibility study a success because of the lessons learned about its implementation, as well as the opportunity to collect information to compare the R2AR against other instruments and outcomes (findings are forthcoming). We learned about the strategies that would enhance a broader study of implementation effectiveness at a future time. Modifiable factors within the study design will enable us to improve implementation and measure additional implementation outcomes, like sustainability, fidelity, satisfaction, acceptability, adoption [22].

## Conclusion

Implementing an activity like administration, completion, and use of a PROM requires clients and providers to alter the way in which they currently behave. In an established system with ascribed roles and statuses, it is incumbent on change agents and researchers to educate on *WHY* the PROM is important [36]. The primary purpose of the R2AR is to empower clients to drive care decisions and enable providers to measure recovery in an objective way through a harm reduction approach [3]. Clients can drive individualized recovery pathways and promote working on the areas that are most important to them. Meanwhile, providers can track empirically how clients are doing in a way that aligns with each patient’s unique definition of recovery and complements a hard reduction approach. A different type of relationship is manifested when stakeholders (e.g., clients and providers) assume new ways of portraying their respective roles in an ascribed system. It is a relationship that supports therapeutic alliance through mutual respect and shared goals [57]. In turn, this changes how the work of recovery is accomplished.

As the substance use treatment system struggles with defining best practices and demonstrating quality of care, PROs offer a remedy to both improving treatment delivery and demonstrating recovery progress [3, 58]. Therefore, studies that test the feasibility of PROMs in clinical practice should hypothesize and test how implementation and quality improvement techniques can address barriers to adoption [22]. For the future, as we learned, it will be critical to test the feasibility and effectiveness of various modalities, (e.g., digital PROM completion) and more generally for remote client monitoring. Future feasibility studies might also consider the flow of remote data collection, such as requesting completion of other instruments at a different time to isolate the analysis of the instrument in question. Changes to treatment delivery will inevitably shape how patient care information is collected and care quality is measured, and we must be ready and willing to adapt to future needs.

## Data Availability

No datasets were generated or analysed during the current study.

## Abbreviations

EHR: Electronic health record
IHI: Institute for Healthcare Improvement
NCM: Nurse care manager
OBAT: Office-based addiction treatment
OUD: Opioid use disorder
PRO: Patient-reported outcome
PROM: Patient-reported outcome measure
QI: Quality improvement
R2AR: Response to Addiction Recovery (a PROM for recovery)
RA: Research assistant
SUD: Substance use disorder
